# Supplementary material for: High-density vertical sidewall MoS2 transistors through T-shape vertical lamination
Source: Nat Commun. 2024 Jul 10;15:5774. doi: 10.1038/s41467-024-50185-4 (PMC11233715; doi:10.1038/s41467-024-50185-4)
Supplement: Supplementary file 1 — Supplementary Information [file 41467_2024_50185_MOESM1_ESM.pdf]

Supplementary Information for

High-density vertical sidewall MoS_2 transistors through T-shape vertical lamination

Quanyang Tao[1,2,4], Ruixia Wu[1,3,4], Xuming Zou[1,*], Yang Chen[1], Wanying Li[1], Zheyi Lu[1], Likuan Ma[1], Lingan Kong[1], Donglin Lu[1], Xiaokun Yang[1], Wenjing Song[1], Wei Li[3], Liting Liu[1], Shuimei Ding[1], Xiao Liu[1], Xidong Duan[3], Lei Liao[2,*], & Yuan Liu[1,*]

[1]Key Laboratory for Micro-Nano Optoelectronic Devices of Ministry of Education, School of Physics and Electronics, Hunan University, Changsha, China.

[2]Changsha Semiconductor Technology and Application Innovation Research Institute, College of Semiconductors (College of Integrated Circuits), Hunan University, Changsha, China.

[3]State Key Laboratory for Chemo/Biosensing and Chemometrics, College of Chemistry and Chemical Engineering, Hunan University, Changsha, China.

[4]These authors contributed equally to this work.

[*]Corresponding author. E-mail: yuanliuhnu@hnu.edu.cn; zouxuming@hnu.edu.cn; liaolei@whu.edu.cn

Supplementary Figures

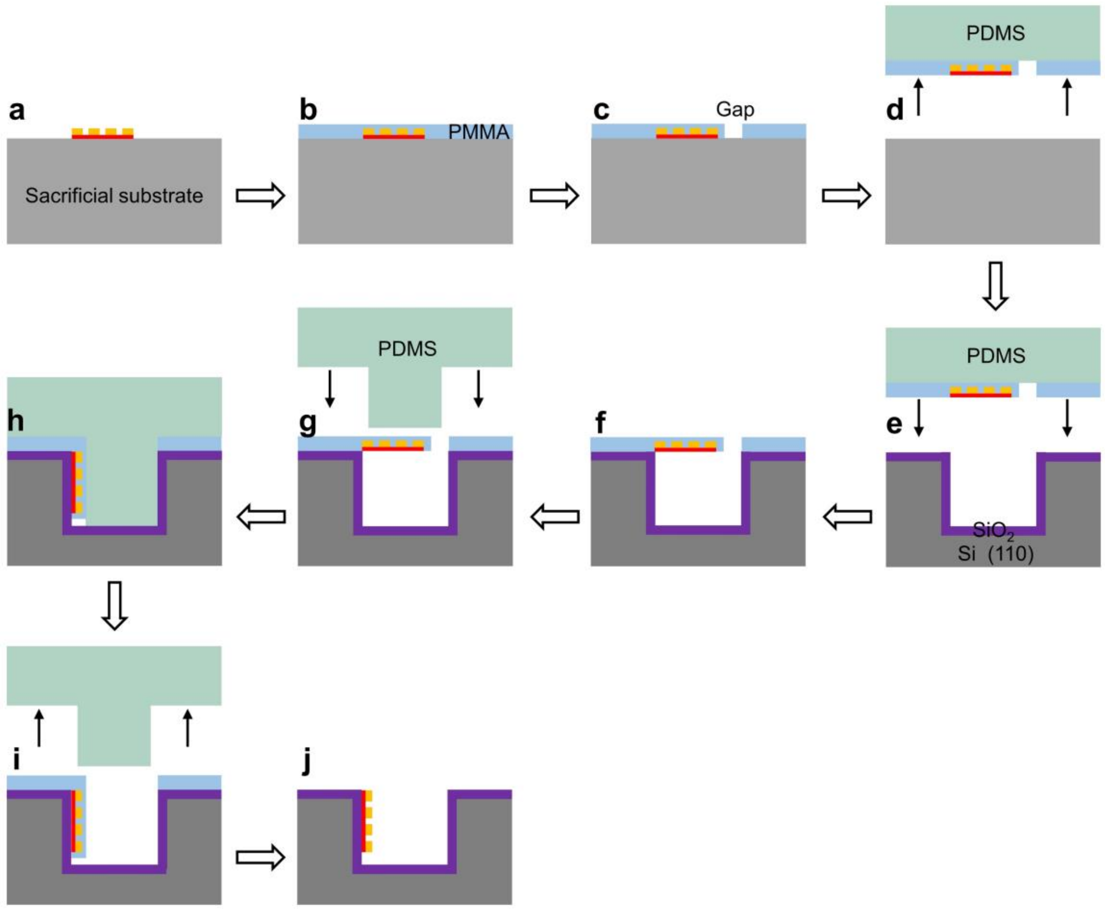

Supplementary Fig. 1. Fabrication processes of vertical sidewall transistors. a–j, Detailed fabrication processes of the vertical lamination with several steps including: MoS_2 transistors pre-fabricated on a sacrificial substrate (**a**); The substrate functionalized by HMDS and sin-coated PMMA layer (**b**); the PMMA gap created by electron-beam lithography and development processes (**c**); device layer mechanically released from the substrate (**d**); device layer dry transferred and suspended on top of a trench structure with the assistance of flat PDMS (**e**, **f**); device layer laminated onto the sidewall using T-shape PDMS (**g**–**i**). MoS_2 vertical transistors after PMMA removal (**j**).

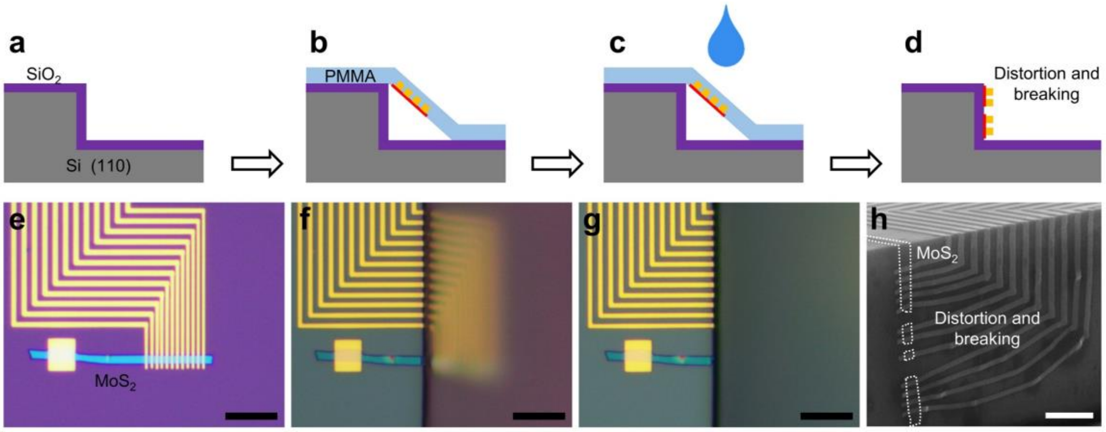

Supplementary Fig. 2. Conventional wet-transfer process for vertical integration. a-d, Schematics of the conventional wet-transfer method for vertical integration with 4 steps: fabrication of vertical Si sidewall by etching (**a**), transfer of MoS_2 transistors on the Si sidewall (**b**), immersion of the sample in ethylene glycol (**c**) and transistors attached to the sidewall after annealing (**d**). **e**–**g**, Optical images of MoS_2 transistors prefabricated on a sacrificial substrate (**e**), physically transferred on top of the Si sidewall (**f**) and attached to the sidewall after annealing (**g**). Scale bars, 10 μm. **h**, SEM image of the MoS_2 vertical transistors, where the MoS_2 transistors were severely deformed and fractured due to large stretching force during the solution evaporation. Scale bar, 5 μm.

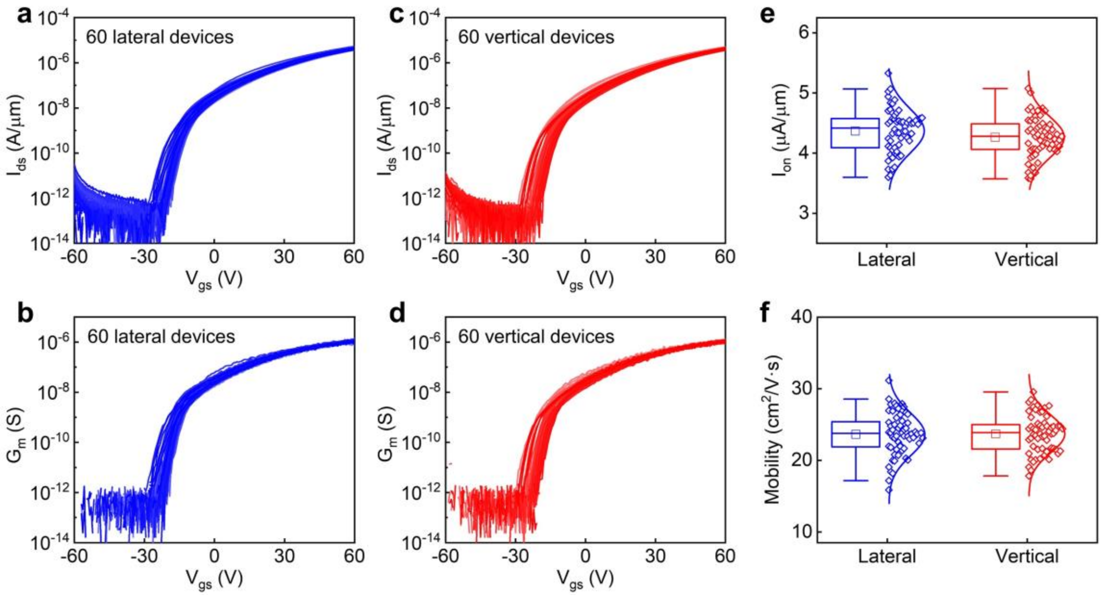

Supplementary Fig. 3. Statistical analysis of MoS_2 transistors performance. **a**, **b**, The transfer characteristics (**a**) and transconductance curves (**b**) of lateral MoS_2 devices at V_{ds}=1 V. **c**, **d**, The transfer characteristics (**c**) and transconductance curves (**d**) of vertical MoS_2 devices at V_{ds}=1 V. **e**, **f**, Statistical distribution of on-state current (**e**) and field effect mobility (**f**) of lateral and vertical MoS_2 devices. Error bars in **e**, **f** are determined from the statistical standard deviations of 60 devices. I_{on}, on-state current; G_m, transconductance.

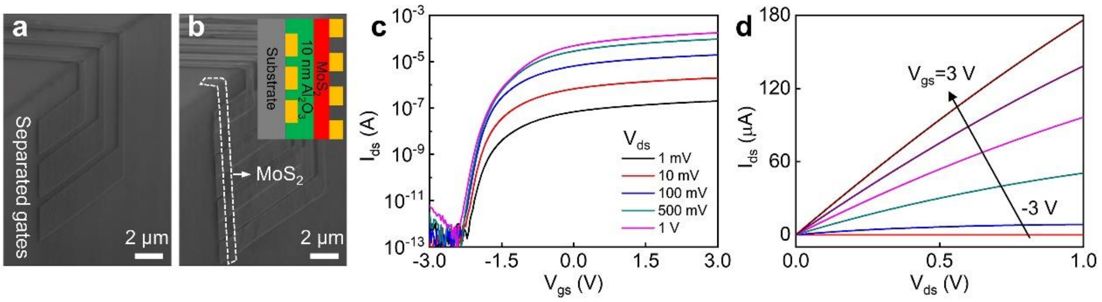

Supplementary Fig. 4. Electrical characteristics of vertical MoS_2 transistors with separated gates. a, SEM image of separated gates on the vertical sidewall. **b**, SEM image of vertical MoS_2 transistors with separated gates. Inset is the cross-sectional schematic of the vertical MoS_2 transistors. **c**, **d**, I_{ds}–V_{gs} transfer characteristics (**c**) and I_{ds}–V_{ds} output curves (**d**) of the vertical MoS_2 transistor with the separated gate.

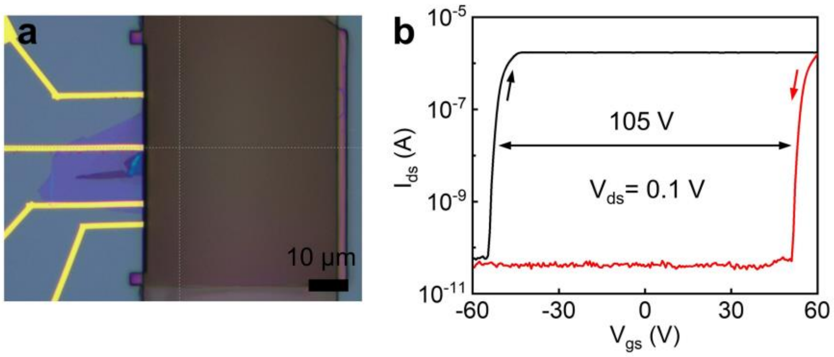

Supplementary Fig. 5. Vertical inverter and memory. a, Optical image of the vertical inverter and memory. **b**, The transfer characteristics of the vertical floating gate memory.

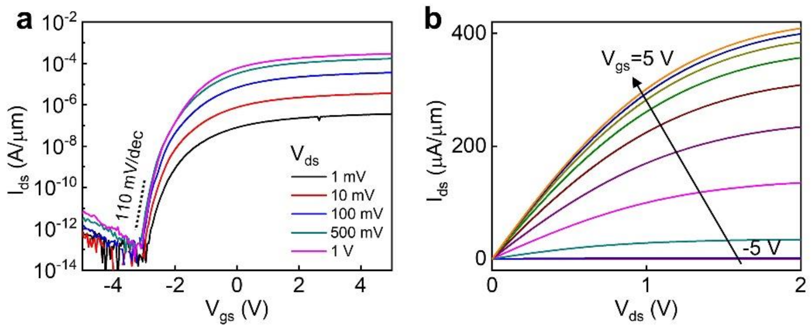

Supplementary Fig. 6. Electrical characteristics of vertical MoS_2 transistor with 150 nm channel length using 10 nm thick Al_2O_3 as side gate dielectric. a, **b**, I_{ds}–V_{gs} transfer characteristics (**a**) and I_{ds}–V_{ds} output curves (**b**) of the vertical MoS_2 transistor using stronger gate control.

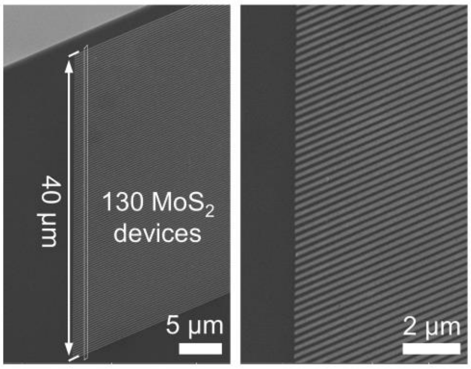

Supplementary Fig. 7. SEM images of 130 vertical devices on a 40 µm deep sidewall with higher device density.

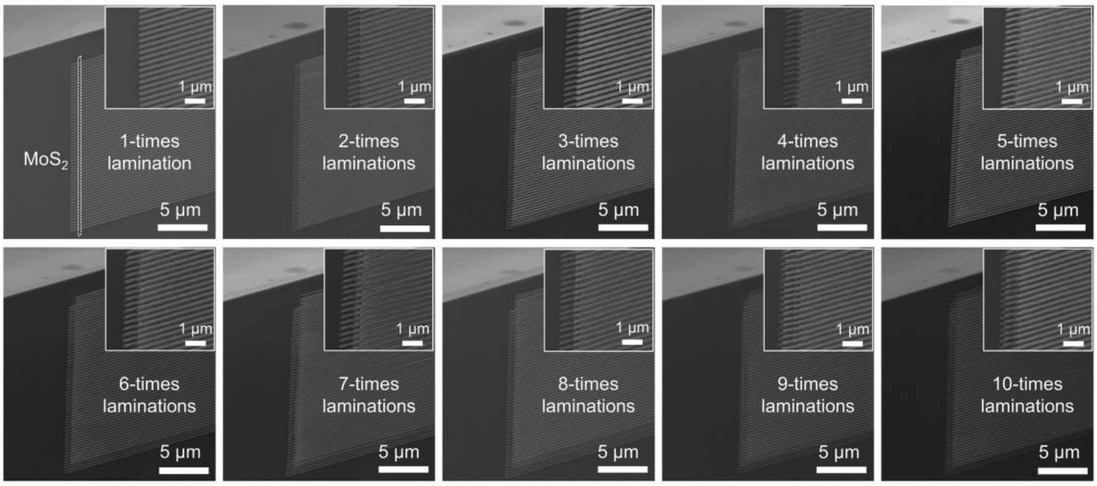

Supplementary Fig. 8. SEM images of vertical devices, from first layer lamination to 10-times laminations.

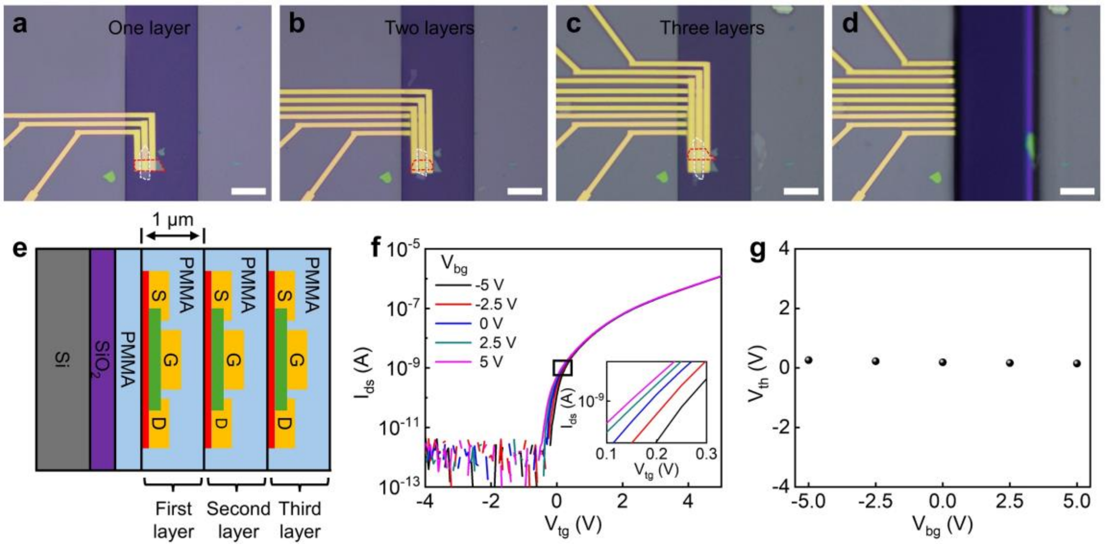

Supplementary Fig. 9. Layer by layer integrated MoS_2 transistors with top gates using BN flakes as the dielectrics. a, Optical image of the suspended first-layer MoS_2 transistor. **b**, Optical image of the suspended two-layer MoS_2 transistors. **c**, Optical image of the suspended three-layer MoS_2 transistors. The red and white dotted boxes are MoS_2 and BN in the first-layer (**a**), second-layer (**b**) and third-layer (**c**) device, respectively. **d**, Optical image of the vertical three-layer MoS_2 transistors. Scale bars are 10 μm. **e**, Cross-sectional schematic of the vertical three-layer devices. **f**, Transfer curves of the second layer MoS_2 transistor under different back gate voltages from -5 V to 5 V, at V_{ds} of 0.1 V. **g**, The threshold voltage as a function of back gate voltage. V_{tg}, top gate voltage; V_{bg}, back gate voltage; V_{th}, threshold voltage.

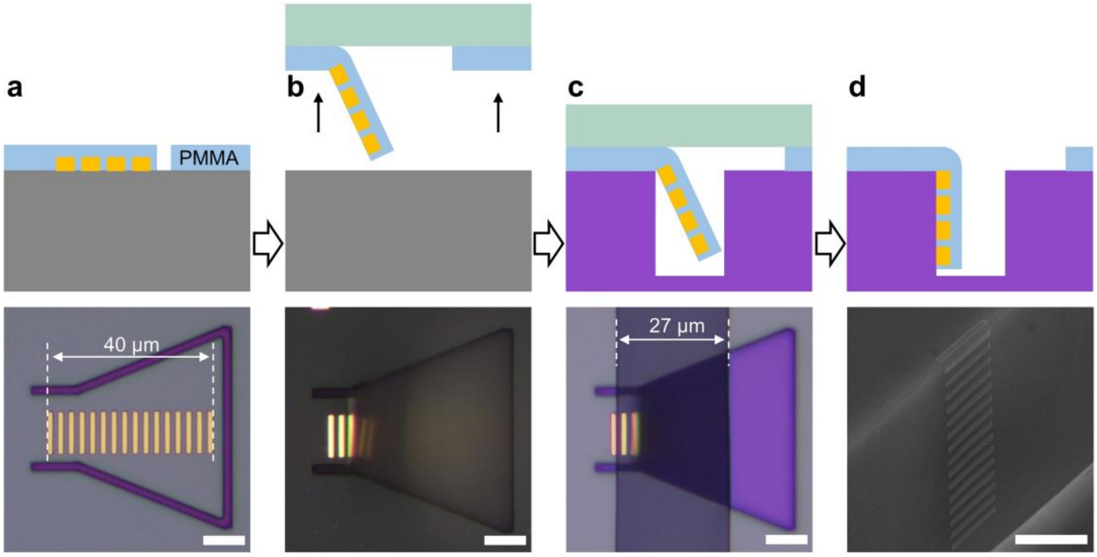

Supplementary Fig. 10. Improvement of vertical lamination process. **a**, Pre-fabricated planar electrodes with patterned PMMA on a sacrificial substrate. **b**, Separation of electrode layer from PDMS after peeling off. **c**, Release the electrodes into the trench. **d**, The electrodes attach to the sidewall after vertical lamination. Scale bars are 10 μm.
